# 2-[(*E*)-2-(4-Methyl­benzene­sulfonamido)ethyl­iminiometh­yl]-4-nitro­phenolate

**DOI:** 10.1107/S1600536809031481

**Published:** 2009-08-15

**Authors:** Marife Tüfekçi, Gökhan Alpaslan, Mustafa Macit, Ahmet Erdönmez

**Affiliations:** a Department of Physics, Faculty of Arts & Science, Ondokuz Mayıs University, TR-55139 Kurupelit–Samsun, Turkey; b Department of Chemistry, Faculty of Arts & Science, Ondokuz Mayıs University, 55139 Samsun, Turkey

## Abstract

The mol­ecule of the title compound, C_16_H_17_N_3_O_5_S, crystallizes in a zwitterionic form, with a strong intra­molecular N—H⋯O hydrogen bond. The dihedral angle between the two benzene rings is 7.06 (9)°. In the crystal, mol­ecules are linked into chains along the *c* axis by inter­molecular N—H⋯O hydrogen bonds.

## Related literature

For general background to Schiff bases, see: Calligaris *et al.* (1972 ▶); Cohen *et al.* (1964 ▶); Hadjoudis *et al.* (1987 ▶); Karabıyık *et al.* (2008 ▶). For the crystal structure of 2-[2-(1*H*-indol-3-yl)ethyl­iminiometh­yl]-4-nitro­phenolate, see: Ali *et al.* (2008 ▶). For hydrogen-bond motifs, see: Bernstein *et al.* (1995 ▶).
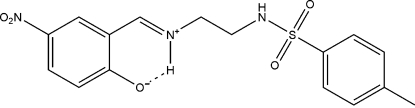


## Experimental

### 

#### Crystal data

C_16_H_17_N_3_O_5_S
*M* *_r_* = 363.39
Monoclinic, 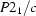

*a* = 17.915 (5) Å
*b* = 7.342 (5) Å
*c* = 13.055 (5) Å
β = 103.928 (5)°
*V* = 1666.7 (14) Å^3^
*Z* = 4
Mo *K*α radiation
μ = 0.23 mm^−1^
*T* = 296 K
0.68 × 0.50 × 0.26 mm

#### Data collection

Stoe IPDS-II diffractometer
Absorption correction: integration (*X-RED32*; Stoe & Cie, 2002 ▶) *T*_min_ = 0.887, *T*_max_ = 0.956
22908 measured reflections
3272 independent reflections
2845 reflections with *I* > 2σ(*I*)
*R*_int_ = 0.024

#### Refinement

*R*[*F* ^2^ > 2σ(*F* ^2^)] = 0.032
*wR*(*F* ^2^) = 0.094
*S* = 1.07
3272 reflections
236 parameters
H atoms treated by a mixture of independent and constrained refinement
Δρ_max_ = 0.26 e Å^−3^
Δρ_min_ = −0.27 e Å^−3^

### 

Data collection: *X-AREA* (Stoe & Cie, 2002 ▶); cell refinement: *X-AREA*; data reduction: *X-RED32*; program(s) used to solve structure: *SHELXS97* (Sheldrick, 2008 ▶); program(s) used to refine structure: *SHELXL97* (Sheldrick, 2008 ▶); molecular graphics: *ORTEP-3 for Windows* (Farrugia, 1997 ▶); software used to prepare material for publication: *WinGX* (Farrugia, 1999 ▶).

## Supplementary Material

Crystal structure: contains datablocks I, global. DOI: 10.1107/S1600536809031481/ci2882sup1.cif

Structure factors: contains datablocks I. DOI: 10.1107/S1600536809031481/ci2882Isup2.hkl

Additional supplementary materials:  crystallographic information; 3D view; checkCIF report

## Figures and Tables

**Table 1:** Hydrogen-bond geometry (Å, °)

*D*—H⋯*A*	*D*—H	H⋯*A*	*D*⋯*A*	*D*—H⋯*A*
N3—H1⋯O3^i^	0.78 (2)	2.06 (2)	2.833 (2)	170 (2)
N2—H1*A*⋯O3	0.87 (2)	1.94 (2)	2.648 (2)	137 (2)

Symmetry code: (i) 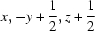.

**Fig. 1.**
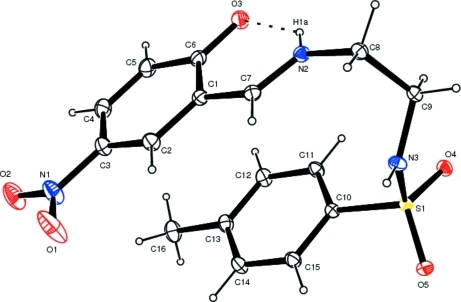
The molecular structure of (I), showing the atom-numbering scheme. Displacement ellipsoids are drawn at the 30% probability level and H atoms are shown as small spheres of arbitrary radii. The dashed line indicates a hydrogen bond.

**Fig. 2.**
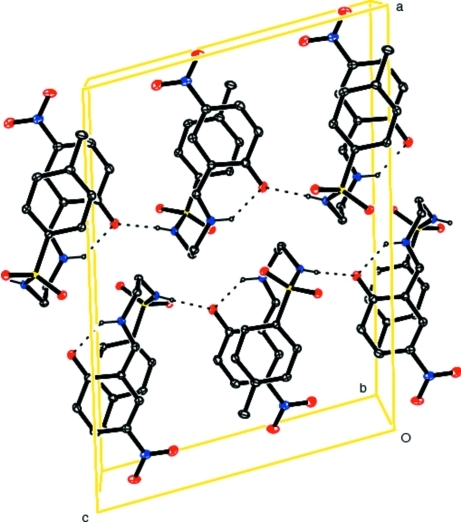
A packing diagram for (I). H atoms not involved in hydrogen bonding (dashed lines) have been omitted for clarity.

## supplementary crystallographic information

### Comment

Schiff bases have been extensively used as ligands in the field of coordination chemistry (Calligaris *et al.*, 1972). Schiff base compounds can be classified by their photochromic and thermochromic characteristics (Cohen *et al.*, 1964). These properties result from a proton transfer from the hydroxyl O atom to the imine N atom (Hadjoudis *et al.*, 1987). Schiff bases exhibit two well known tautomeric forms *viz*. OH and NH tautomers, and they also exist in zwitterionic form (Karabıyık *et al.*, 2008). Our investigations show that the title compund has a zwitterionic form with a strong intramolecular N—H···O hydrogen bond (Fig. 1).

The C7N2 [1.292 (2) Å] and C6—03 [1.272 (2) Å] bond distances in the title compound are comparable to those [1.292 (2) and 1.264 (2) Å] observed in a related zwitterionic structure (Ali *et al.*, 2008). The molecule adopts a folded conformation. The dihedral angle between the two benzene rings is 7.06 (9)°.

The intramolecular N2—H1A···O3 hydrogen bond generates an S(6) ring motif (Bernstein *et al.*, 1995). The molecules are linked into chains (Fig. 2) along the *c* axis by intermolecular N—H···O hydrogen bonds (Table 1).

### Experimental

2-Hydroxy-5-nitrobenzaldehyde (10 mg, 5.98× 10^-2^ mmol) in ethanol (20 ml) was added to a solution of *N*-*p*-tolyl-sulfonylethylenediamine (12.7 mg, 5.98× 10^-2^ mmol) in ethanol (20 ml). The reaction mixture was stirred for 1 h under reflux. Single crystals of the title compound were obtained by slow evaporation of an ethylacetate solution (yield 55%; m.p. 446–447 K).

### Refinement

Atoms H1 and H1A were located in a difference map and were refined freely. The remaining H atoms were placed in calculated positions and constrained to ride on their parent atoms, with C—H = 0.93–0.97 Å and *U*_iso_(H) = 1.2*U*_eq_(C) or 1.5*U*_eq_(methyl C).

### Crystal data

**Table:**

C_16_H_17_N_3_O_5_S	*F*(000) = 760
*M**_r_* = 363.39	*D*_x_ = 1.448 Mg m^−^^3^
Monoclinic, *P*2_1_/*c*	Mo *K*α radiation, λ = 0.71069 Å
Hall symbol: -P 2ybc	Cell parameters from 37649 reflections
*a* = 17.915 (5) Å	θ = 1.6–28.0°
*b* = 7.342 (5) Å	µ = 0.23 mm^−^^1^
*c* = 13.055 (5) Å	*T* = 296 K
β = 103.928 (5)°	Block, yellow
*V* = 1666.7 (14) Å^3^	0.68 × 0.50 × 0.26 mm
*Z* = 4	

### Data collection

**Table:**

Stoe IPDS-II diffractometer	3272 independent reflections
Radiation source: fine-focus sealed tube	2845 reflections with *I* > 2σ(*I*)
graphite	*R*_int_ = 0.024
Detector resolution: 6.67 pixels mm^-1^	θ_max_ = 26.0°, θ_min_ = 2.3°
ω scans	*h* = −22→22
Absorption correction: integration (*X-RED32*; Stoe & Cie, 2002)	*k* = −9→9
*T*_min_ = 0.887, *T*_max_ = 0.956	*l* = −16→15
22908 measured reflections	

### Refinement

**Table:**

Refinement on *F*^2^	Secondary atom site location: difference Fourier map
Least-squares matrix: full	Hydrogen site location: inferred from neighbouring sites
*R*[*F*^2^ > 2σ(*F*^2^)] = 0.032	H atoms treated by a mixture of independent and constrained refinement
*wR*(*F*^2^) = 0.094	*w* = 1/[σ^2^(*F*_o_^2^) + (0.0542*P*)^2^ + 0.2378*P*] where *P* = (*F*_o_^2^ + 2*F*_c_^2^)/3
*S* = 1.07	(Δ/σ)_max_ = 0.004
3272 reflections	Δρ_max_ = 0.26 e Å^−^^3^
236 parameters	Δρ_min_ = −0.27 e Å^−^^3^
0 restraints	Extinction correction: *SHELXL97* (Sheldrick, 2008), Fc^*^=kFc[1+0.001xFc^2^λ^3^/sin(2θ)]^-1/4^
Primary atom site location: structure-invariant direct methods	Extinction coefficient: 0.0025 (8)

### Special details

**Table:**

Geometry. All e.s.d.'s (except the e.s.d. in the dihedral angle between two l.s. planes) are estimated using the full covariance matrix. The cell e.s.d.'s are taken into account individually in the estimation of e.s.d.'s in distances, angles and torsion angles; correlations between e.s.d.'s in cell parameters are only used when they are defined by crystal symmetry. An approximate (isotropic) treatment of cell e.s.d.'s is used for estimating e.s.d.'s involving l.s. planes.
Refinement. Refinement of *F*^2^ against ALL reflections. The weighted *R*-factor *wR* and goodness of fit *S* are based on *F*^2^, conventional *R*-factors *R* are based on *F*, with *F* set to zero for negative *F*^2^. The threshold expression of *F*^2^ > σ(*F*^2^) is used only for calculating *R*-factors(gt) *etc*. and is not relevant to the choice of reflections for refinement. *R*-factors based on *F*^2^ are statistically about twice as large as those based on *F*, and *R*- factors based on ALL data will be even larger.

### Fractional atomic coordinates and isotropic or equivalent isotropic displacement parameters (Å^2^)

**Table:**

	*x*	*y*	*z*	*U*_iso_*/*U*_eq_	
C1	0.73857 (9)	0.1439 (2)	0.58535 (12)	0.0464 (3)	
C2	0.81399 (10)	0.1345 (2)	0.64584 (14)	0.0559 (4)	
H2	0.8238	0.1405	0.7190	0.067*	
C3	0.87338 (10)	0.1163 (3)	0.59823 (15)	0.0604 (4)	
C4	0.86008 (11)	0.1075 (3)	0.48830 (16)	0.0648 (5)	
H4	0.9012	0.0958	0.4567	0.078*	
C5	0.78743 (11)	0.1160 (3)	0.42812 (15)	0.0642 (5)	
H5	0.7796	0.1093	0.3552	0.077*	
C6	0.72214 (10)	0.1346 (2)	0.47198 (13)	0.0523 (4)	
C7	0.67882 (9)	0.1617 (2)	0.63841 (13)	0.0463 (3)	
H7	0.6925	0.1648	0.7118	0.056*	
C8	0.54749 (9)	0.1973 (2)	0.65034 (13)	0.0466 (4)	
H8A	0.5661	0.1505	0.7214	0.056*	
H8B	0.5024	0.1275	0.6162	0.056*	
C9	0.52559 (8)	0.3947 (2)	0.65549 (12)	0.0438 (3)	
H9A	0.5085	0.4419	0.5843	0.053*	
H9B	0.4829	0.4035	0.6889	0.053*	
C10	0.69671 (8)	0.65737 (19)	0.62312 (11)	0.0400 (3)	
C11	0.69016 (9)	0.6244 (2)	0.51721 (12)	0.0495 (4)	
H11	0.6420	0.6182	0.4705	0.059*	
C12	0.75608 (10)	0.6008 (3)	0.48147 (13)	0.0571 (4)	
H12	0.7515	0.5781	0.4102	0.068*	
C13	0.82838 (10)	0.6100 (2)	0.54833 (15)	0.0553 (4)	
C14	0.83365 (10)	0.6421 (3)	0.65479 (15)	0.0601 (4)	
H14	0.8818	0.6474	0.7015	0.072*	
C15	0.76879 (9)	0.6661 (2)	0.69225 (13)	0.0522 (4)	
H15	0.7733	0.6882	0.7636	0.063*	
C16	0.89969 (12)	0.5917 (3)	0.50703 (19)	0.0795 (6)	
H16A	0.8863	0.5403	0.4374	0.119*	
H16B	0.9359	0.5136	0.5529	0.119*	
H16C	0.9223	0.7097	0.5045	0.119*	
N1	0.95110 (10)	0.1062 (3)	0.66326 (18)	0.0917 (6)	
N2	0.60663 (7)	0.17413 (18)	0.59235 (11)	0.0456 (3)	
N3	0.58876 (7)	0.50612 (18)	0.71366 (10)	0.0448 (3)	
O1	0.96069 (10)	0.1084 (5)	0.75840 (16)	0.1571 (12)	
O2	1.00420 (9)	0.0954 (4)	0.62016 (16)	0.1183 (7)	
O3	0.65360 (7)	0.1427 (2)	0.41569 (9)	0.0669 (4)	
O4	0.55334 (6)	0.75149 (15)	0.58460 (9)	0.0479 (3)	
O5	0.63776 (6)	0.81294 (15)	0.76080 (9)	0.0522 (3)	
S1	0.614192 (19)	0.69488 (5)	0.67146 (3)	0.03887 (13)	
H1	0.6088 (10)	0.478 (2)	0.7710 (14)	0.048 (5)*	
H1A	0.5970 (12)	0.176 (3)	0.5235 (17)	0.067 (6)*	

### Atomic displacement parameters (Å^2^)

**Table:**

	*U*^11^	*U*^22^	*U*^33^	*U*^12^	*U*^13^	*U*^23^
C1	0.0470 (8)	0.0456 (8)	0.0471 (8)	−0.0002 (6)	0.0121 (7)	−0.0028 (7)
C2	0.0522 (9)	0.0660 (10)	0.0494 (9)	−0.0005 (8)	0.0124 (7)	0.0003 (8)
C3	0.0452 (9)	0.0709 (11)	0.0658 (11)	0.0025 (8)	0.0148 (8)	0.0017 (9)
C4	0.0601 (11)	0.0720 (12)	0.0701 (12)	0.0056 (9)	0.0307 (9)	−0.0012 (10)
C5	0.0709 (12)	0.0753 (12)	0.0507 (9)	0.0086 (10)	0.0232 (9)	−0.0062 (9)
C6	0.0568 (10)	0.0516 (9)	0.0487 (9)	0.0038 (7)	0.0130 (7)	−0.0072 (7)
C7	0.0505 (9)	0.0449 (8)	0.0432 (8)	−0.0021 (6)	0.0107 (7)	−0.0007 (6)
C8	0.0438 (8)	0.0475 (8)	0.0502 (9)	−0.0058 (6)	0.0147 (7)	0.0030 (7)
C9	0.0348 (7)	0.0498 (8)	0.0472 (8)	−0.0037 (6)	0.0109 (6)	0.0008 (7)
C10	0.0389 (7)	0.0409 (7)	0.0399 (7)	−0.0016 (6)	0.0089 (6)	0.0033 (6)
C11	0.0425 (8)	0.0643 (10)	0.0407 (8)	−0.0008 (7)	0.0079 (6)	0.0049 (7)
C12	0.0556 (10)	0.0750 (12)	0.0440 (9)	0.0037 (8)	0.0185 (7)	0.0086 (8)
C13	0.0469 (9)	0.0583 (10)	0.0651 (11)	0.0003 (7)	0.0222 (8)	0.0094 (8)
C14	0.0378 (8)	0.0749 (12)	0.0637 (11)	−0.0013 (8)	0.0044 (7)	0.0005 (9)
C15	0.0444 (8)	0.0643 (10)	0.0450 (8)	0.0001 (7)	0.0054 (7)	−0.0027 (7)
C16	0.0553 (11)	0.0987 (16)	0.0943 (15)	0.0034 (11)	0.0372 (11)	0.0117 (13)
N1	0.0490 (9)	0.1412 (19)	0.0847 (13)	0.0029 (11)	0.0154 (9)	0.0064 (13)
N2	0.0471 (7)	0.0463 (7)	0.0444 (7)	−0.0015 (5)	0.0131 (6)	−0.0026 (6)
N3	0.0466 (7)	0.0521 (8)	0.0328 (7)	−0.0071 (6)	0.0041 (5)	0.0045 (6)
O1	0.0575 (10)	0.327 (4)	0.0791 (12)	0.0105 (15)	0.0021 (9)	0.0096 (18)
O2	0.0494 (8)	0.195 (2)	0.1165 (14)	0.0089 (11)	0.0310 (9)	−0.0005 (14)
O3	0.0600 (8)	0.0888 (9)	0.0472 (7)	0.0093 (7)	0.0038 (6)	−0.0159 (6)
O4	0.0417 (5)	0.0488 (6)	0.0494 (6)	0.0043 (4)	0.0034 (5)	0.0055 (5)
O5	0.0512 (6)	0.0543 (6)	0.0504 (6)	−0.0036 (5)	0.0108 (5)	−0.0159 (5)
S1	0.03696 (19)	0.0403 (2)	0.0382 (2)	0.00009 (14)	0.00693 (14)	−0.00238 (14)

### Geometric parameters (Å, °)

**Table:**

C1—C2	1.393 (2)	C10—C15	1.387 (2)
C1—C7	1.414 (2)	C10—S1	1.7631 (15)
C1—C6	1.440 (2)	C11—C12	1.381 (2)
C2—C3	1.362 (2)	C11—H11	0.93
C2—H2	0.93	C12—C13	1.378 (3)
C3—C4	1.399 (3)	C12—H12	0.93
C3—N1	1.448 (3)	C13—C14	1.390 (3)
C4—C5	1.350 (3)	C13—C16	1.507 (2)
C4—H4	0.93	C14—C15	1.376 (2)
C5—C6	1.427 (2)	C14—H14	0.93
C5—H5	0.93	C15—H15	0.93
C6—O3	1.272 (2)	C16—H16A	0.96
C7—N2	1.292 (2)	C16—H16B	0.96
C7—H7	0.93	C16—H16C	0.96
C8—N2	1.4529 (19)	N1—O1	1.212 (3)
C8—C9	1.507 (2)	N1—O2	1.219 (2)
C8—H8A	0.97	N2—H1A	0.87 (2)
C8—H8B	0.97	N3—S1	1.5976 (16)
C9—N3	1.4537 (19)	N3—H1	0.777 (18)
C9—H9A	0.97	O4—S1	1.4325 (11)
C9—H9B	0.97	O5—S1	1.4329 (12)
C10—C11	1.381 (2)		
			
C2—C1—C7	118.18 (15)	C10—C11—C12	119.13 (15)
C2—C1—C6	120.72 (15)	C10—C11—H11	120.4
C7—C1—C6	121.10 (15)	C12—C11—H11	120.4
C3—C2—C1	120.26 (16)	C13—C12—C11	121.97 (16)
C3—C2—H2	119.9	C13—C12—H12	119.0
C1—C2—H2	119.9	C11—C12—H12	119.0
C2—C3—C4	120.93 (17)	C12—C13—C14	117.95 (15)
C2—C3—N1	118.96 (18)	C12—C13—C16	121.15 (18)
C4—C3—N1	120.11 (17)	C14—C13—C16	120.87 (17)
C5—C4—C3	119.81 (16)	C15—C14—C13	121.12 (16)
C5—C4—H4	120.1	C15—C14—H14	119.4
C3—C4—H4	120.1	C13—C14—H14	119.4
C4—C5—C6	122.64 (17)	C14—C15—C10	119.75 (16)
C4—C5—H5	118.7	C14—C15—H15	120.1
C6—C5—H5	118.7	C10—C15—H15	120.1
O3—C6—C5	122.91 (16)	C13—C16—H16A	109.5
O3—C6—C1	121.46 (15)	C13—C16—H16B	109.5
C5—C6—C1	115.63 (16)	H16A—C16—H16B	109.5
N2—C7—C1	124.73 (15)	C13—C16—H16C	109.5
N2—C7—H7	117.6	H16A—C16—H16C	109.5
C1—C7—H7	117.6	H16B—C16—H16C	109.5
N2—C8—C9	111.47 (12)	O1—N1—O2	122.7 (2)
N2—C8—H8A	109.3	O1—N1—C3	118.65 (18)
C9—C8—H8A	109.3	O2—N1—C3	118.7 (2)
N2—C8—H8B	109.3	C7—N2—C8	122.67 (14)
C9—C8—H8B	109.3	C7—N2—H1A	114.1 (14)
H8A—C8—H8B	108.0	C8—N2—H1A	123.0 (14)
N3—C9—C8	112.72 (13)	C9—N3—S1	123.98 (11)
N3—C9—H9A	109.0	C9—N3—H1	118.3 (13)
C8—C9—H9A	109.0	S1—N3—H1	117.4 (13)
N3—C9—H9B	109.0	O4—S1—O5	119.20 (8)
C8—C9—H9B	109.0	O4—S1—N3	107.39 (7)
H9A—C9—H9B	107.8	O5—S1—N3	107.26 (8)
C11—C10—C15	120.07 (14)	O4—S1—C10	107.82 (7)
C11—C10—S1	120.62 (11)	O5—S1—C10	106.12 (7)
C15—C10—S1	119.29 (12)	N3—S1—C10	108.74 (7)
			
C7—C1—C2—C3	179.67 (16)	C12—C13—C14—C15	−0.7 (3)
C6—C1—C2—C3	0.0 (3)	C16—C13—C14—C15	177.35 (18)
C1—C2—C3—C4	0.2 (3)	C13—C14—C15—C10	0.3 (3)
C1—C2—C3—N1	−179.76 (18)	C11—C10—C15—C14	0.1 (3)
C2—C3—C4—C5	−0.4 (3)	S1—C10—C15—C14	−178.20 (13)
N1—C3—C4—C5	179.6 (2)	C2—C3—N1—O1	2.3 (4)
C3—C4—C5—C6	0.3 (3)	C4—C3—N1—O1	−177.7 (3)
C4—C5—C6—O3	180.00 (19)	C2—C3—N1—O2	−177.8 (2)
C4—C5—C6—C1	0.0 (3)	C4—C3—N1—O2	2.2 (4)
C2—C1—C6—O3	179.83 (16)	C1—C7—N2—C8	−178.27 (14)
C7—C1—C6—O3	0.2 (3)	C9—C8—N2—C7	96.71 (17)
C2—C1—C6—C5	−0.1 (2)	C8—C9—N3—S1	131.22 (13)
C7—C1—C6—C5	−179.75 (16)	C9—N3—S1—O4	14.65 (14)
C2—C1—C7—N2	179.17 (15)	C9—N3—S1—O5	143.88 (12)
C6—C1—C7—N2	−1.2 (3)	C9—N3—S1—C10	−101.76 (13)
N2—C8—C9—N3	−63.94 (17)	C11—C10—S1—O4	−21.41 (15)
C15—C10—C11—C12	−0.1 (2)	C15—C10—S1—O4	156.87 (13)
S1—C10—C11—C12	178.14 (13)	C11—C10—S1—O5	−150.18 (13)
C10—C11—C12—C13	−0.3 (3)	C15—C10—S1—O5	28.09 (15)
C11—C12—C13—C14	0.7 (3)	C11—C10—S1—N3	94.72 (14)
C11—C12—C13—C16	−177.38 (18)	C15—C10—S1—N3	−87.00 (14)

### Hydrogen-bond geometry (Å, °)

**Table:**

*D*—H···*A*	*D*—H	H···*A*	*D*···*A*	*D*—H···*A*
N3—H1···O3^i^	0.78 (2)	2.06 (2)	2.833 (2)	170 (2)
N2—H1A···O3	0.87 (2)	1.94 (2)	2.648 (2)	137 (2)

Symmetry codes: (i) *x*, −*y*+1/2, *z*+1/2.
